# ANP32B deficiency impairs proliferation and suppresses tumor progression by regulating AKT phosphorylation

**DOI:** 10.1038/cddis.2016.8

**Published:** 2016-02-04

**Authors:** S Yang, L Zhou, P T Reilly, S-M Shen, P He, X-N Zhu, C-X Li, L-S Wang, T W Mak, G-Q Chen, Y Yu

**Affiliations:** 1 Key Laboratory of Cell Differentiation and Apoptosis of Chinese Ministry of Education, Rui-Jin Hospital, Shanghai Jiao-Tong University School of Medicine (SJTU-SM), Shanghai, China; 2 Department of Surgery, Branch of Shanghai First People's Hospital, SJTU-SM, Shanghai, China; 3 Laboratory of Inflammation Biology, National Cancer Centre Singapore, Singapore, Singapore; 4 State Key Laboratory of Genetic Engineering, Minhang Hospital, Fudan University, Shanghai, China; 5 Campbell Family Cancer Research Institute, University Health Network, Toronto, ON, Canada

## Abstract

The acidic leucine-rich nuclear phosphoprotein 32B (ANP32B) is reported to impact normal development, with *Anp32b-*knockout mice exhibiting smaller size and premature aging. However, its cellular and molecular mechanisms, especially its potential roles in tumorigenesis, remain largely unclear. Here, we utilize 'knockout' models, RNAi silencing and clinical cohorts to more closely investigate the role of this enigmatic factor in cell proliferation and cancer phenotypes. We report that, compared with *Anp32b* wild-type (*Anp32b*^+/+^) littermates, a broad panel of tissues in *Anp32b-*deficient (*Anp32b*^−/−^) mice are demonstrated hypoplasia. *Anp32b*^−/−^ mouse embryo fibroblast cell has a slower proliferation, even after oncogenic immortalization. *ANP32B* knockdown also significantly inhibits *in vitro* and *in vivo* growth of cancer cells by inducing G_1_ arrest. In line with this, ANP32B protein has higher expression in malignant tissues than adjacent normal tissues from a cohort of breast cancer patients, and its expression level positively correlates with their histopathological grades. Moreover, *ANP32B* deficiency downregulates AKT phosphorylation, which involves its regulating effect on cell growth. Collectively, our findings suggest that ANP32B is an oncogene and a potential therapeutic target for breast cancer treatment.

The acidic leucine-rich nuclear phosphoprotein 32 kDa (ANP32) protein family are characterized by a N-terminal leucine-rich repeat domain and a C-terminal low-complexity acidic region.^1^ In mammals, the ANP32 family has at least three members named ANP32A, ANP32B and ANP32E, and they regulate a wide spectrum of biological processes including chromatin regulation,^2, 3, 4, 5, 6^ caspase activation,^7, 8, 9^ protein phosphatase inhibition^10, 11, 12^ and intracellular transport.^13, 14^ Although early investigations suggested that three ANP32 members functionally overlap,^10^ they are reported to have diverse roles in cancer progression. *ANP32A* was shown to inhibit cell transformation^15, 16, 17^ and has reduced expression in prostate and breast cancer.^18, 19^ *ANP32E* was reported to have enhanced expression in gastric cancer,^20^ and a high expression of *ANP32E* was associated with better survival rate in follicular lymphoma.^21^ Previously we reported that *ANP32B*, also designated as PHAPI2 or SSP29, is a negative prognostic indicator for human breast cancer.^22^ Full analysis of the expression and functional role of *ANP32B* in cancer progression has still not been undertaken.

Knockout mouse studies demonstrated that loss of *Anp32b*, but not *Anp32a* and *Anp32e*, caused a high degree of perinatal lethality and reduced body weight,^22, 23, 24, 25^ indicating a greater importance of *Anp32b* in normal development. In addition, gene expression analysis indicates that elevated *ANP32B* mRNA expression correlates with highly proliferative tissues.^22^ We also showed that *ANP32B* acts as a negative regulator of leukemic cell apoptosis,^8^ and inhibits all-*trans* retinoic acid induced leukemic cell differentiation.^26, 27^ Although these studies strongly suggested *ANP32B* as a master regulator of cell fate determination, its cellular and molecular mechanisms are still not understood. Considering that some physiological and pathological processes share many common molecular regulators,^28^ and *ANP32B* mRNA expression is a marker for aggressive breast cancer,^22^ we proposed that ANP32B also functions in breast cancer. Here, we used Anp32b-knockout mice, multiple breast cancer cell lines and clinical patient samples to uncover the potential role for ANP32B in cell proliferation of both mouse embryo fibroblasts (MEFs) and breast cancer cells, and find that loss of ANP32B by knockout or RNAi silencing reduced rates of cell proliferation. We also show that RNAi silencing induces an extended G_1_-phase of the cell cycle. In addition, phosphorylation of AKT, an upstream regulator of cell cycle-associated proteins, is lower coincident with reduced ANP32B upon silencing and in both mouse and human cancers.

## Results

### Anp32b^−/−^ MEFs are impaired in cell proliferation and oncogenic transformation

**Figure 1:**
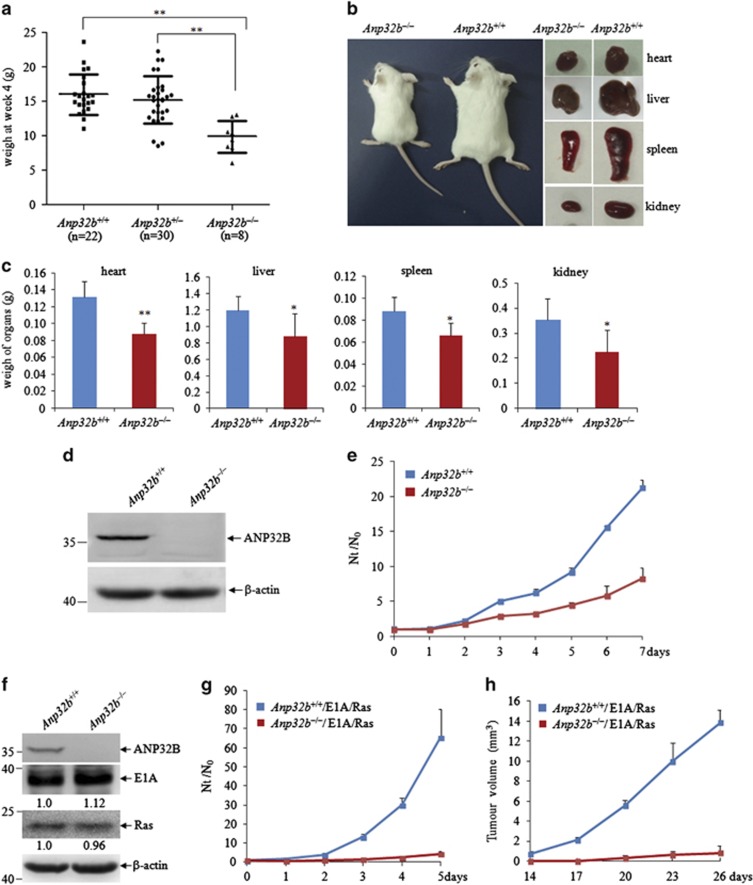
*Anp32b* deficiency impairs normal cell proliferation and oncogenic transformation. (**a**) The body weight of 22 *Anp32b* ^+/+^, 30 *Anp32b* ^+/−^ and 8 *Anp32b* ^−/−^ mice at 4 weeks of age. Data were analyzed using Mann–Whitney *U*-test. **P<0.01. (**b**) Photographs of appearance and organs of *Anp32b*^−/−^ mice and *Anp32b*^+/+^ littermate at 4 months of age. (**c**) The weight of organs from *Anp32b*^+/+^ and *Anp32b*^−/−^ mice at 4 months of age. Data are presented as mean±S.D. and significance is *P<0.05 (*n*=4). (**d**) Western blots for the indicated protein in primary *Anp32b*^+/+^ and *Anp32b*^−/−^ MEF cells. (**e**) Proliferation of primary *Anp32b*^+/+^ and *Anp32b*^−/−^ MEFs was monitored for the indicated times. N_t_/N_0_ represents the cell number at a given time normalized to the cell number at day 0. Data are presented as mean±S.D. The experiment is representative of three separate experiments. (**f**) Western blots for the indicated protein in immortalized *Anp32b*^+/+^ and *Anp32b*^−/−^ MEF cells. (**g**) Proliferation of immortalized *Anp32b*^+/+^ and *Anp32b*^−/−^ MEFs was monitored for the indicated times. Data are presented as mean±S.D. (**h**) Immortalized MEF cells were subcutaneously injected into nude mice and the size of masses were analyzed. Data are presented as mean±S.D.

As seen in mixed-bred homozygous *Anp32b*^−/−^ mice,^22^ the Balb/c-congenic *Anp32b*^−/−^ mice also had a statistically significant reduction of body weight compared with wild-type (*Anp32b*^+/+^) and heterozygous (*Anp32b*^+/−^) mice at 4 weeks after birth (Figure 1a). Here we also found the decreased weigh of the *Anp32b* ^−/−^ mice was accompanied by the reduced size of organs such as the heart, liver, spleen and kidney (Figures 1b and c). To determine whether this is due to reduced cell volumes or hypoplasia, histological H&E staining and flow cytometric evaluation were performed. The results showed that there was no obvious cell size difference between these organs in *Anp32b*^−/−^ and *Anp32b*^+/+^ mice (Supplementary Figure S1A and B). However, cell numbers in organs such as spleen and thymus were dramatically decreased in *Anp32b*^−/−^ mice (Supplementary Figure S1C), supporting that *Anp32b* deficiency causes a hypoplastic phenotype in multiple organs.

To functionally characterize the role of *Anp32b* in normal cell proliferation, we isolated MEFs from *Anp32b*^+/+^ and *Anp32b*^−/−^ mice. The ANP32B protein was totally knocked out in *Anp32b*^−/−^ MEF cells (Figure 1d). Cell proliferation assay showed that primary *Anp32b*^−/−^ MEF cells divided significantly more slowly than *Anp32b*^+/+^ MEF cells (Figure 1e). Given the effect of *Anp32b* on cell proliferation, we set out to assess whether *Anp32b* deficiency could inhibit oncogenic transformation. To this end, MEFs were immortalized by infection with a retrovirus encoding two oncogenes, adenovirus 5 E1A and constitutively active form of H-RasV12 (Figure 1f). The results showed that the immortalized *Anp32b*^−/−^ MEF cells also presented lower cell proliferation than *Anp32b*^+/+^ MEF cells (Figure 1g). We also subcutaneously injected immortalized *Anp32b*^+/+^ and *Anp32b*^−/−^ MEF cells into nude mice. Mice xenografted with *Anp32b*^−/−^ MEF cells developed significant smaller tumors compared with the mice injected with *Anp32b*^+/+^ MEF cells (Figure 1h). All these data suggest a functional role of *Anp32b* in the proliferation of normal and transformed cells.

### ANP32B knockdown inhibits breast cancer cell proliferation *in vitro*

**Figure 2:**
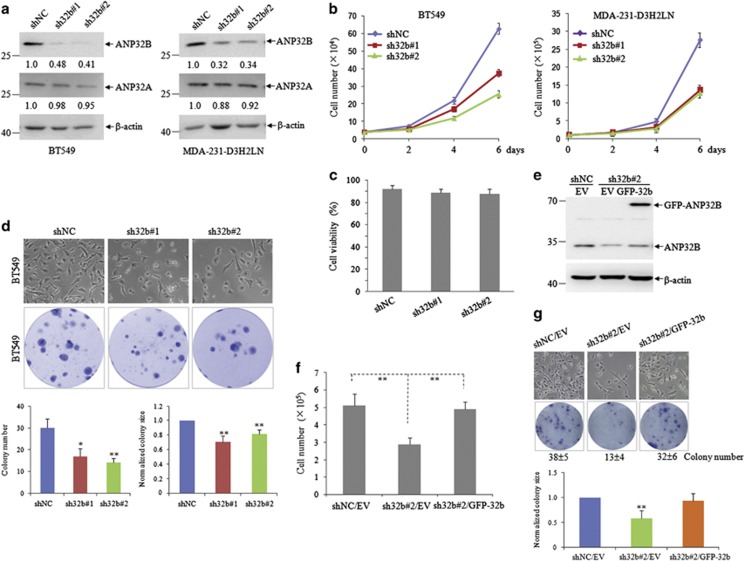
Knockdown of *ANP32B* inhibits breast cancer cells proliferation. (**a**) Breast cancer BT549, MDA-231-D3H2LN cells were stably infected with shNC and sh32b, and the indicated proteins were detected by western blot with β-actin as a loading control. (**b**) Cell counting of shNC- and sh32b-infected BT549, MDA-231-D3H2LN cells after 2, 4 and 6 days of growth. (**c**) Cell viability after 6 days of growth was measured by trypan blue exclusion. Data are presented as mean± S.D. of triplicate in an independent experiment, which was repeated for more than three times. (**d**) The morphology of shNC- and sh32b-infected BT549 cells under phase contrast microscopy (upper). Influence of *ANP32B* on colony formation of BT549 cells. Representative dishes are presented (middle). The number and size of clones were calculated for each well of six-well plates and shown in the *y* axis in the bottom panel. Data are presented as mean± S.D. and significance is *P<0.05, **P<0.01, which was repeated for more than three times. (**e**) ShNC- and sh32b-infected breast cancer MDA-231-D3H2LN cells were stably transfected with empty vector (EV) and GFP-tagged ANP32B, followed by immunoblots for the indicated proteins. (**f**) Cell counting of shNC/EV, sh32b/EV and sh32b/GFP-ANP32B MDA-231-D3H2LN cells after 3 days of growth. Data are presented as mean± S.D. and significance is **P<0.01, which was repeated for more than three times. (**g**) Representative images from the morphology and colony formation of shNC/EV, sh32b/EV and sh32b/GFP-ANP32B MDA-231-D3H2LN cells

We further investigated whether *ANP32B* regulates cancer cell proliferation with breast cancer cells as models. For this purpose, we used two pairs of shRNAs (sh32b#1 and sh32b#2) specifically against *ANP32B* to generate stable *ANP32B* knockdown along with a control shRNA transfectant (shNC) in BT549, MCF7 and MDA-231-D3H2LN breast cancer cell lines. These two specific shRNAs could effectively knockdown *ANP32B* but not its closely related *ANP32A* expression in these breast cancer cell lines (Figure 2a and Supplementary Figure S2A). Then, we examined the effect of *ANP32B* knockdown on breast cancer cell proliferation. As shown in Figures 2b and c, *ANP32B* knockdown significantly inhibited the growth of BT549 cells with no effect on their viability. Similar effects could also be seen in MDA-231-D3H2LN (Figure 2b) and MCF7 cells (Supplementary Figure S2B and C). Compared with the control cells, in addition, BT549 and MCF7 cells with *ANP32B* silencing showed markedly decreased colony formation ability with reduced colony number and size (*P*<0.05; Figure 2d and Supplementary Figure S2D and E). To demonstrate that cell growth inhibition is specifically due to the silencing of *ANP32B*, we re-introduced GFP-tagged *ANP32B* into sh32b#2-transfected MDA-231-D3H2LN cells, and found that re-expression of *ANP32B* could reverse *ANP32B* knockdown-induced cell growth inhibition (Figures 2e and g). Taken together, these data suggest that *ANP32B* may be closely associated with the proliferation of breast cancer cell lines.

### ANP32B promotes cell cycle progression

**Figure 3:**
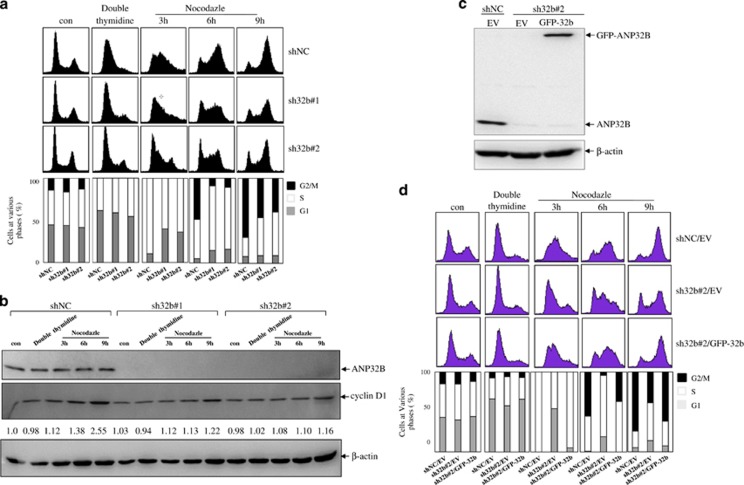
*ANP32B* deficiency induces cell cycle G1/S arrest. (**a**) ShNC- and sh32b-infected BT549 cells were pretreated with thymidine twice and then treated with nocodazole for indicated times. DNA content of treated cells was analyzed by flow cytometry. (**b**) Equal amounts of the corresponding cell lysates were blotted for ANP32B, cyclin D1 and *β*-actin. (**c**) ShNC- and sh32b-infected breast cancer BT549 cells were stably transfected with empty vector (EV) and GFP-tagged ANP32B, followed by immunoblots for the indicated proteins. (**d**) ShNC/EV, sh32b/EV and sh32b/GFP-ANP32B BT549 cells were pretreated with thymidine twice and then treated with nocodazole for indicated times. DNA content of treated cells was analyzed by flow cytometry

To examine whether the effect of *ANP32B* on breast cancer cell proliferation is partly due to cell cycle arrest, we used a double thymidine block to synchronize cells at the G_1_/S border, followed by addition of nocodazole to block cells in G_2_/M. Flow cytometry analysis was then used to monitor the progression of cells from G_1_/S to G_2_/M. The results showed that after nocodazole treatment within 3 h 89.5% of cells in shNC BT549 cells entered the S phase, whereas cells at the S phase only had 59.9 and 63.8%, respectively, in sh32b#1 and sh32b#2-infected cells. After nocodazole treatment for 9 h, only 46.3 and 39.8% of total cells entered the G_2_/M phase in two sh32b-infected BT549 cells compared with 69.6% of total cells in shNC BT549 clones (Figure 3a). In addition, we analyzed the cell cycle regulatory proteins. As shown in Figure 3b, cyclin D1 protein level had no alteration between NC and sh32b-infected cells. However, cyclin D1 was time-dependently increased in shNC-infected BT549 cells upon nocodazole treatment, which was remarkably inhibited in sh32b-infected cells. Similarly, we re-introduced *ANP32B* into sh32B#2-transfected BT549 cells (Figure 3c). Cell cycle analysis showed that complementation by *ANP32B* could rescue the cell cycle G_1_ phase arrest in sh32b BT549 cells (Figure 3d). Collectively, these results indicate that *ANP32B* promotes cell cycle progression at the G_1_ phase.

### Loss of ANP32B suppresses breast tumor growth *in vivo*

**Figure 4:**
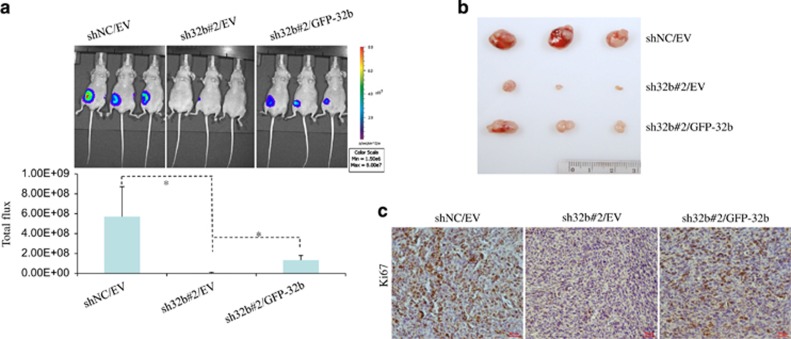
Re-expression of *ANP32B* promotes breast cancer cell growth *in vivo.* MDA-231-D3H2LN cells infected with shNC/EV, sh32b/EV and sh32b/GFP-ANP32B were injected into right side of the mammary fat pad of the mice. (**a**) After 4 weeks, the tumor growth was monitored using bioluminescent imaging and the representative bioluminescent images (top)/total flux (bottom) are shown. Data are presented as mean± S.D. and symbol *indicates *P*<0.05 between lined groups. (**b**) The macroscopic appearances of tumors at 4 weeks after injection. (**c**) Representative images of immunohistochemical staining for Ki-67 of mammary tumors at 4 weeks after injection

These *in vitro* results prompted us to examine whether *ANP32B* has some effects on breast tumor growth *in vivo*. Hence, shNC, sh32b#2 and sh32b#2/GFP-*ANP32B*-infected MDA-231-D3H2LN cell line (Figure 2e), which was derived from breast cancer cell line MDA-MB-231 with stable luciferase expression, were injected into the mammary fat pad of nude mice. Luciferase photon fluxes were monitored and the representative tumors are shown (Figures 4a and b). Consistent with the *in vitro* findings, reduction of *ANP32B* led to a significant inhibition of tumor size at the fourth week after injection, which could be partially reversed by re-expression of *ANP32B* (Figures 4a and b). Furthermore, *ANP32B* knockdown tumor cells showed obviously weaker Ki-67 staining compared with control tumor cells, which could be also reversed by re-expression of *ANP32B*, suggesting that ANP32B knockdown indeed decreased cell proliferation *in vivo* (Figure 4c). All these data strongly suggested that specific loss of *ANP32B* could significantly inhibit breast cancer growth *in vivo*.

### ANP32B is highly expressed in human breast cancer

**Figure 5:**
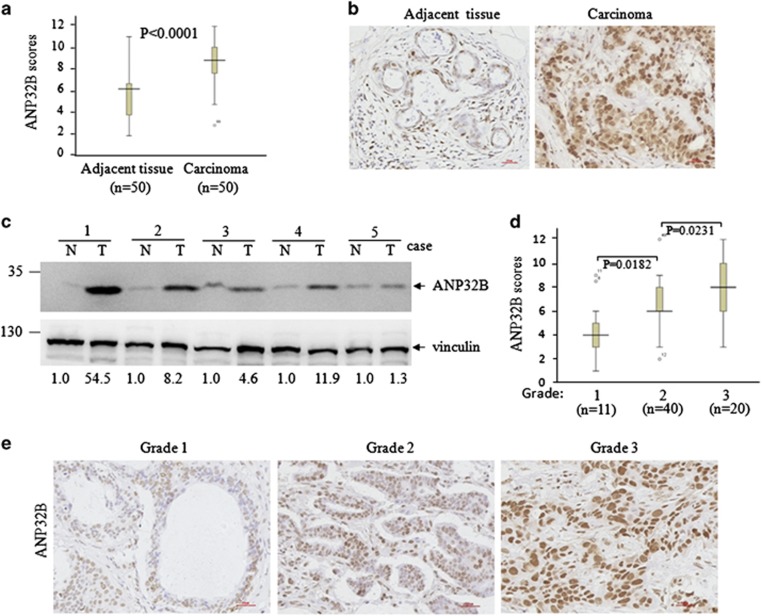
ANP32B expression is elevated in breast cancer tumors and positive correlates with historical grade of breast cancers. (**a**) ANP32B expression was plotted using the immunohistochemical scores as described in the Material and Methods. ANP32B expression scores are shown as box plots, with the horizontal lines representing the median; the bottom and top of the boxes representing the 25th and 75th percentiles, respectively; and vertical bars representing the range of data. We compared breast cancer tumors with matched adjacent normal breast epithelium using the Mann–Whitney test, *n*=100. (**b**) Representative images from immunohistochemical staining of ANP32B from one pair of breast cancer and adjacent normal tissues. The scale bar represents 30 *μ*m. (**c**) Expression of ANP32B in five pairs of clinical breast cancer specimens. N and T mean adjacent normal tissue and paired breast cancer tumor, respectively. (**d**) Box plots of ANP32B expression in breast cancers with different historical grades. Data were analyzed by one-way ANOVA test. (**e**) Representative images from immunohistochemical staining of ANP32B from three cases in different histological grades (1–3). The scale bar represents 30 *μ*m

Previously, we examined the relationship between *ANP32B* mRNA expression and breast cancer patient prognosis using information from three available data sets and reported that patients whose tumors showed the highest *ANP32B* mRNA levels had significant shorter survival.^22^ Here we performed Immunohistochemical staining (IHC) staining on 50 breast tumor tissues and the matched adjacent normal tissues, and found that breast tumor tissues presented higher ANP32B expression compared with adjacent normal tissues (Figures 5a and b). Moreover, an increase of ANP32B protein level in tumor tissues over adjacent normal tissues was also confirmed by western blot analysis in five paired clinical breast cancer specimens (Figure 5c). These data indicate that ANP32B expression is enhanced in human breast cancer at the protein level.

We next evaluated the correlation between ANP32B expression and clinicopathological parameters. As presented in Supplementary Figure S3, there was no significant correction for ANP32B expression with age or clinical stage of breast cancer patients. However, ANP32B was associated significantly with histological grade. Higher levels of ANP32B was correlated with higher histological grade (I *versus* II; *P*=0.0182, II *versus* III; *P*=0.0231) (Figure 5d). Figure 5e depicts three representative IHC images respectively for low, medium and high ANP32B expressions of cancer tissues with different histological grade. These data suggest that elevated ANP32B protein expression in breast cancer is directly related with histological grade of cancer tissues.

### ANP32B has positive correlation with p-AKT and regulates AKT activation

**Figure 6:**
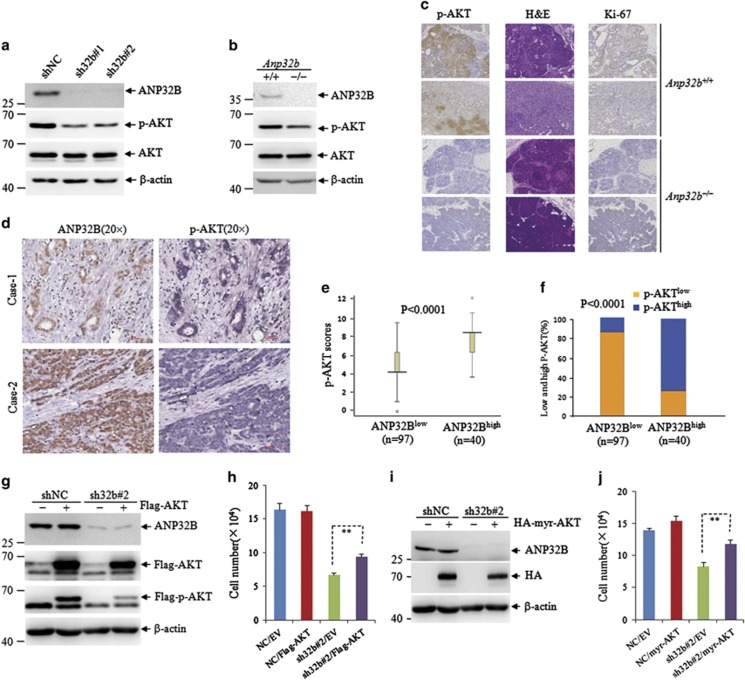
The effects of *ANP32B* on the AKT activation and the correlation of ANP32B and p-AKT expression in breast cancer patients. (**a**) The expression of AKT and the phosphorylation of AKT in shNC- and sh32b-infected BT549 cells. (**b**) The expression of AKT and the phosphorylation of AKT in *Anp32b*^+/+^ and *Anp32b* ^−/−^ MEF cells. (**c**) H&E staining and immunohistochemical analysis were used to determine the level of phosphorylation of AKT and Ki-67 expression in mammary tumors from DMBA-induced *Anp32b*^+/+^ and *Anp32b*^−/−^ mice. (**d**) Representative IHC images of breast cancer samples for the indicated proteins. The scale bar represents 30 *μ*m. (**e**–**f**) Box plots of p-AKT scores (**e**) and the percentage of tumors with high and low p-AKT expressions (**f**) in those with high and low ANP32B expressions. (**g**) ShNC- and sh32b-infected breast cancer BT549 cells were stably transfected with empty vector (EV) and Flag-AKT, followed by immunoblots for the indicated proteins. (**h**) ShNC- and sh32b-infected breast cancer BT549 cells were stably transfected with empty vector (EV) and HA-myr-AKT, followed by immunoblots for the indicated proteins. (**i**) Cell counting of EV- and Flag-AKT-transfected BT549 cells after 3 days of growth. Data are presented as mean± S.D. and significance is **P<0.01, which was repeated for more than three times. (**j**) Cell counting of EV- and HA-myr-AKT-transfected BT549 cells after 3 days of growth. Data are presented as mean± S.D. and significance is **P<0.01, which was repeated for more than three times

We analyzed the expressions of cyclins such as cyclin D1/3, cyclin-dependent kinases (CDKs) including CDK4, CDK6, CDK2, CDK inhibitor p27, as well as ERK and P38 in ANP32B silencing BT549 and MDA-231-D3H2LN cells. The results showed that knockdown of *ANP32B* failed to change all these protein levels (Supplementary Figure S4). More interestingly, *ANP32B* knockdown significantly reduced the phosphorylated AKT at Ser473 rather than AKT protein (Figure 6a). Of note, it did not change phosphorylated ERK and P38 (Supplementary Figure S4). A similar impact of *ANP32B* on AKT phosphorylation was evident in *Anp32b*^−/−^ MEF cells (Figure 6b). In line with this, a carcinogen 7,12-dimethylbenz(a)anthracene (DMBA)-induced mammary tumors^30^ derived from *Anp32b* ^−/−^ but not *Anp32b* ^+/+^ mice also displayed negative p-AKT staining expression (Figure 6c).

We detected ANP32B and p-AKT expression by IHC staining in tumor tissues from breast cancer patients. Figure 6d depicts two representative IHC images for ANP32B and p-AKT expression. To better understand the correlation of ANP32B and p-AKT, we stratified the cohort into two groups based on ANP32B staining (ANP32B^low^ and ANP32B^high^), and P-AKT was scored using either median expression score (Figure 5e) or the percentage of low and high expression scores (Figure 5f). The results demonstrated a highly positive correlation between the ANP32B and P-AKT.

### ANP32B regulates breast cancer cell proliferation through AKT activation

To further investigate the contribution of AKT signaling to the role of *ANP32B* in cell proliferation, we ectopically expressed AKT in *ANP32B* knockdown cells to evaluate whether it might overcome the suppression effect of *ANP32B* deficiency on cell proliferation. BT549 cells were stably co-transfected with shNC or sh32b#2 together with vector or flag-AKT, and the results showed that the p-AKT level was increased but still lower in sh32b cells compared with shNC BT549 cells (Figure 6g). As expected, the ectopically expressed AKT could rescue *ANP32B* knockdown-induced cell growth inhibition in BT549 cells (Figure 6h). Considering that Akt overexpression-restored pAkt levels might be responsible for reversion of effects in ANP32B knockdown cells, the HA-myr-AKT with constitutive activation of AKT^31^ was re-expressed in shNC and sh32b#2 BT549 cells (Figure 6i). Consistent with data in Figure 6h, the enforced expression of HA-myr-AKT could also rescue *ANP32B* knockdown-induced cell growth inhibition in BT549 cells (Figure 6j). All these results indicated that AKT signaling mediated *ANP32B* knockdown-induced cell growth inhibition.

## Discussion

In this study, we used three models to examine the role of ANP32B in cell proliferation and oncogenesis. The knockout mouse model demonstrated that ANP32B has a broad impact on cell proliferation evidenced by hypoplasia in many organs, and that loss of ANP32B inhibits normal cell proliferation and suppresses transformation. *ANP32B* silencing by RNAi also inhibited breast cancer cell proliferation *in vitro* and *in vivo*. Thus, ANP32B is an important proliferation-related nuclear protein. Our further investigation with synchronize cells at the G_1_/S border, followed by addition of nocodazole to block cells in G_2_/M showed that ANP32B silencing significantly retarded the progression of cells from G_1_/S to G_2_/M.

Clinical data set analyses showed that ANP32B protein level is highly expressed in breast cancer patients and the elevated ANP32B protein expression is directly related with histological grade of breast cancer tissues. These data suggested that ANP32B acts as a predictive indicator in breast cancer treatment. However, owing to the limitation of patients sample information, relationships between ANP32B overexpression and clinical prognosis were not fully analyzed. Increased ANP32B in tumors and knockdown models also correlated with high p-AKT expression, indicating a possible mechanism through which ANP32B exerts its effect on cell proliferation and tumor progression.

The activated AKT pathway has been demonstrated to have an essential role in normal cell and breast cancer cell proliferation.^32, 33, 34, 35, 36^ We found that the p-AKT level was significantly decreased in *ANP32B* knockdown cells. Furthermore, the restoration of AKT or constitutively active AKT expression could rescue the inhibition of cell proliferation by ANP32B deficiency, suggesting the inhibition of cell proliferation by ANP32B deficiency is primarily mediated through AKT activation in breast cancer cells. How ANP32B might regulate the AKT activation is still unknown. Previous studies have reported that AKT activation could be regulated by many genes, including PH domain leucine-rich repeat protein phosphatase,^37^ serine/threonine protein phosphatase 2A (PP2A),^38^ pyruvate dehydrogenase kinase, isozyme 1 (ref. 39) and phosphatase and tensin homolog (PTEN),^40^ but our preliminary experiments showed that ANP32B failed to interact with PP2A and PTEN (data not shown). So future experiments will be needed to investigate the detailed mechanism about how ANP32B regulates AKT activation.

Totally, our results concluded that ANP32B, through its positive regulation of p-AKT, serves as a master enforcer of cell proliferation. In the physiological context, knockout of ANP32B impedes the proper mammalian development, whereas in the pathological context, ANP32B deficiency functions as a suppressor of tumor growth and transformation. Notably, ANP32B has been highly detected in breast cancer patients, thus highlighting ANP32B as a potential therapeutic target for breast cancer treatment.

## Materials and Methods

### Cell lines and cell culture

Human breast cancer cell lines BT549 and MCF7 were obtained from the cell bank of the Chinese Academy of Sciences (Shanghai, China). MCF7 were cultured in Dulbecco's modified Eagle's medium (Hyclone, Logan, UT, USA) with 10% FBS and 0.01 mg/ml human recombinant insulin. BT549 was maintained in RPMI 1640 (Hyclone) with 10% FBS. The cell line MDA-231-D3H2LN (Xenogen, Alameda, CA, USA) was propagated in Minimum essential medium with Earl's balanced salts solution (Hyclone) medium supplemented with 10% FBS, 1% non-essential amino acids (Hyclone) and 1% sodium pyruvate (Hyclone). Primary MEFs were prepared from littermate *Anp32b*^+/+^ and *Anp32b*^−/−^ E14.5 embroys. For transformed MEFs, primary MEFs were infected with a retrovirus generated from pLPC E1A/ras_v12_ using published techniques.^29^ All cells were fostered in a humidified atmosphere of 5% CO_2_.

### Patients

Fifty pairs of formalin-fixed and paraffin-embedded specimens of breast cancer and adjacent normal tissues were purchased from Shanghai Outdo Biotech Co (Shanghai, China). Detailed information is described in the Supplementary Table S1. We obtained formalin-fixed and paraffin-embedded tumor specimens of breast cancer patients, which were histopathologically diagnosed during January 2003 and June 2010 in the Department of Surgery, Shanghai First People's Branch Hospital. All tumors were primary and were untreated before surgery. Detailed information is described in the Supplementary Table S2. In addition, we also collected five pairs of breast cancer and adjacent normal tissue specimens from Rui-Jin Hospital affiliated to Shanghai Jiao-Tong University School of Medicine for analyzing ANP32B protein expression. These studies were approved by the Medical Ethical Committee of the Affiliated Hospitals, Shanghai First People's Branch Hospital and Rui-Jin Hospital, respectively, and informed consent was obtained from all subjects or their relatives.

### IHC

The protein expression levels of ANP32B and p-AKT were analyzed by IHC with anti-ANP32B and anti-p-AKT polyclonal antibody. All of the staining was assessed by pathologists who were blinded to the origin of the samples and subject outcome. Each specimen was assigned a score according to the intensity of the nucleus, cytoplasmic and/or membrane staining (no staining=0; weak staining=1, moderate staining=2, strong staining=3) and the area extent of stained cells (0%=0, 1–24%=1, 25–49%=2, 50–74%=3, 75–100%=4). The final immunoreactive score (IRS) was determined by multiplying the intensity score with the extent score of stained cells, ranging from 0 (the minimum score) to 12 (the maximum score). Scores of ANP32B and p-AKT were divided into two classifications: low (IRS⩽6) and high (IRS>6). Detailed information of the two cohorts was shown in Supplementary Table S3.

### Plasmids, siRNA designs and transfections

Human *ANP32B* cDNA was cloned and inserted into pBabepuro Vector (Clontech, Mountain View, CA, USA) with GFP tag. Two pairs of complementary siRNA oligonucleotides against *ANP32B* and a pair of scrambled negative control siRNA were synthesized by Invitrogen (Carlsbad, CA, USA), annealed and ligated into pSIREN-RetroQ vector (Clontech). The target sequences for *ANP32B* were 5′-TGACTACCGAGAGAGTGTC-3′ and 5′-GCGAAATAAACAGTTACTC-3′. Constitutively active AKT (HA-myr-AKT) and Flag-AKT were a kind gift from Dr. Yu Jianxiu in Shanghai Jiao-Tong University School of Medicine. Retrovirus was generated by transient transfection of the 293T cell line with FuGENE9 transfection reagent (Roche, Basel, Switzerland). After 48 h of transfection, the viral supernatant was harvested and used for infection of target cells. Stable retroviral transduction was achieved by infection for 48 h, after which selection with either puromycin (1.5 *μ*g/ml) was initiated. Selection was stopped as soon as the non-infected control cell died off, and the media were replaced with normal-growing media.

### Western blots

For the protein expression analysis, standard western blotting was carried out with the following antibodies used: Rabbit polyclonal antibodies against phospho-AKT(Ser473), AKT, phospho-Rb(S780), ERK1, phospho-ERK1, phospho-P38, vinculin, rabbit monoclonal antibodies against Cyclin D1, CDK2, CDK4, P27 and mouse monoclonal antibodies against, CyclinD3, CDK6, β-actin (Cell Signaling, Beverly, MA, USA), goat antibody against ANP32A, P38 (Santa Cruz Biotech, Santa Cruz, CA, USA), rabbit antibody against ANP32B (Proteintech Group, Chicago, IL, USA), E1A polyclonal antibody (Abcam, Cambridgeshire, UK) and H-Ras polyclonal antibody (Signalway, College Park, MD, USA).

### Cell proliferation and colony formation assay

MEF cells proliferation was evaluated by the CCK-8 assay (WST-8; Cell counting kit-8 from Dojindo, Kumamoto, Japan). In brief, each well was pulsed by addition of 10 *μ*l WST-8 for 2 h. Absorbance readings at a wavelength of 450nm were taken on Synergy H4 Hybrid Microplate Reader. Breast cancer cells were plated on 6-cm dishes and were counted every 2 days. Cells were stained with a 0.4% trypan blue solution and counted with a hemacytometer. For colony formation assay, 500 cells were placed in complete growth media and allowed to grow until visible colonies formed in a fresh six-well plate (2 weeks). Cell colonies were fixed with cold methanol, stained with 0.1% crystal violet for 30 min, washed, air dried, photographed and counted.

### Cell cycle analysis

For cell cycle analysis, all these BT549-transfected cells were pretreated with 2 mM thymidine twice to synchronize cells at G1/S border, and then treated with 100 ng/ml nocodazole to block cells in G2/M for indicated times. To analyze cellular DNA content by flow cytometry, 10^6^ cells were collected, rinsed and fixed overnight with 75% cold ethanol at −20 °C. Cells were then treated with 100 *μ*g/ml RNase A in Tris-HCl buffer (pH 7.4) and stained with 25 *μ*g/ml propidium iodide. Samples were then subjected to the analysis by flow cytometry (FACSCalibur, BD Biosciences, San Jose, CA, USA) using CellQuest Pro software (BD Biosciences). Ten thousand cells were acquired and analyzed for the DNA content.

### Animal experiments

Six-week-old female BALB/c nude mice were obtained from Shanghai SLAC Laboratory Animal Co., Shanghai, China. We subcutaneously injected 2 × 10^6^ tumor cell lines into left abdominal mammary fat pad. Starting 4 weeks post injection, the tumor size was monitored weekly by bioluminescence imaging. Mice were anesthetized each time and given intraperitoneal injection of d-luciferin (150 *μ*g/g body weight prepared in phosphate-buffered saline), and 10–15 min after the injection, bioluminescence images were captured with a charge-coupled device camera (IVIS; Xenogen). Mice were manipulated and housed according to protocols approved by Shanghai Medical Experimental Animal Care Commission. For the carcinogen DMBA treatment, virgin female *Anp32b*^+/+^ and *Anp32b*^−/−^ mice were treated with 1 mg doses of DMBA via oral gavage weekly for a total of 6 weeks. Mice were checked by palpation for tumor formation after completion of the DMBA treatments.

### Statistical analysis

All the statistical analyses were performed by the statistical package for social science (SPSS) (v.13) (SPSS Institute). The Mann–Whitney test was used to analyze the differences in different ANP32B expression groups. The *χ*^2^-test was used to evaluate the correlation between ANP32B expression and p-AKT expression. The *P*-values for comparison between line-linked groups were obtained by Student's *t*-test. All statistical tests were two-sided, and *P*<0.05 was considered to be statistically significant.

## Abbreviations

ANP32: acidic leucine-rich nuclear phosphoprotein 32
BLI: bioluminescence imaging
CCK-8: cell counting kit-8
CDK: cyclin-dependent kinase
DMBA: 7,12-dimethylbenz(a)anthracene
MEFs: mouse embryo fibroblasts
FBS: fetal bovine serum
GFP: green fluorescence protein
IHC: immunohistochemical staining
IRS: immunoreactive score
PHAPI2: putative HLA-DR-associated protein I-2
PHLPP: PH domain leucine-rich repeat protein phosphatase
PDK1: pyruvate dehydrogenase kinase isozyme 1
PP2A: protein phosphatase 2A
PTEN: phosphatase and tensin homolog
SSP29: silver-stainable protein 29
