# Supplementary material for: ANP32B deficiency impairs proliferation and suppresses tumor progression by regulating AKT phosphorylation
Source: Cell Death Dis. 2016 Feb 4;7(2):e2082–. doi: 10.1038/cddis.2016.8 (PMC4849165; doi:10.1038/cddis.2016.8)
Supplement: Supplementary Information [file cddis20168x1.docx]

**SUPPLEMENTAL INFORMATION**

**
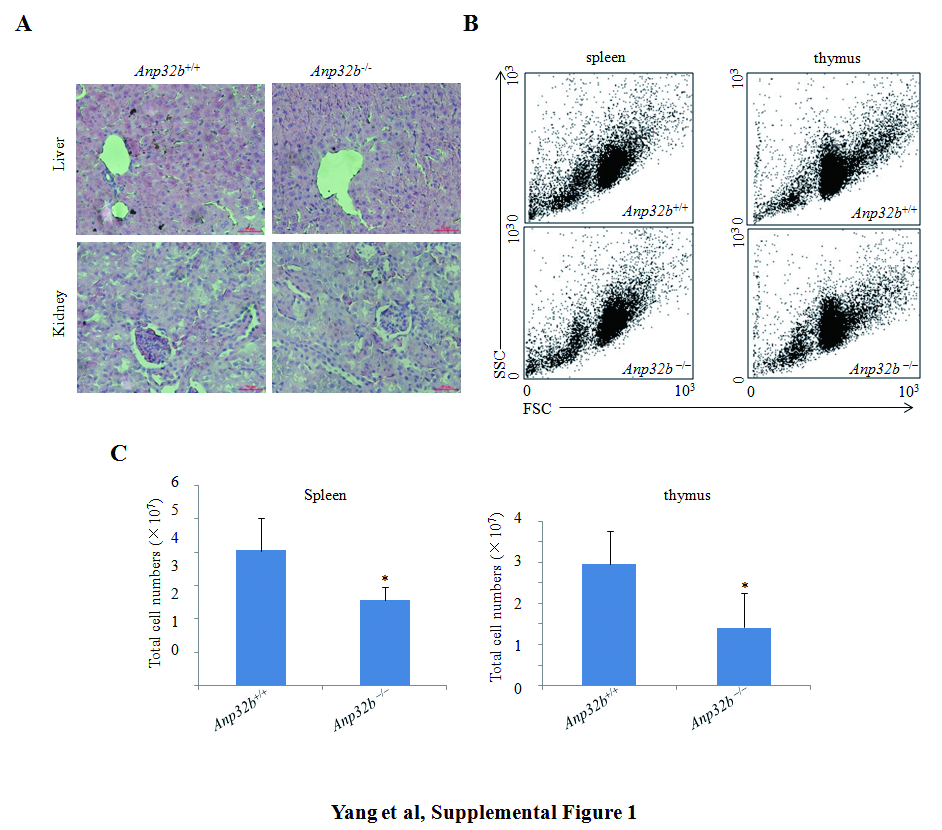
**

Figure S1, related to Figure 1

1. Histopathologic sections were stained with H&E for liver and kidney in *ANP32b*^+/+^ and *ANP32b* ^−/−^ mice.
2. Scattergram of FSC versus SSC was created in flow ctyometry to show the cell size of thymocyte and splenocyte in *ANP32b* ^+/+^ and *ANP32b* ^−/−^ mice.
3. Absolute cell numbers in *ANP32b*^+/+^ and *ANP32b*^−/−^ thymus and spleen. Data are presented as mean±S.D. and significance is *p<0.05 (n=4).

`


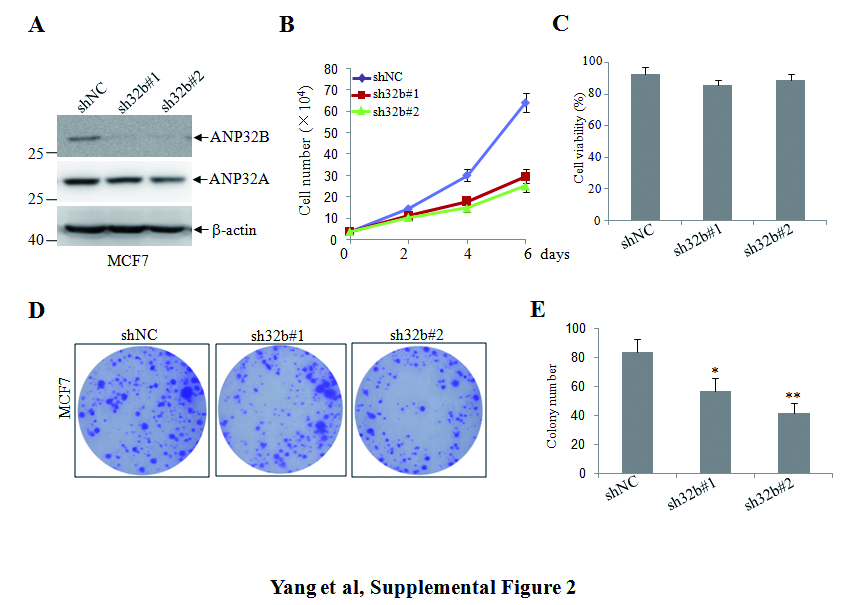


Figure S2, related to Figure 2

(A) Breast cancer MCF7 cells were stably infected with shNC and sh32b, and the indicated proteins were detected.

(B) Cell counting of shNC and sh32b-infected MCF7 cells after 2, 4 and 6 days of growth. Data are presented as mean±S.D, which was repeated for more than three times.

(C) Cell viability after 6 days of growth was measured by trypan-blue exclusion.

(D) Influence of *ANP32B* on colony formation of MCF7 cells. Representative dishes are presented.

(E) The number of clones were counted for each well of six-well plates and shown in the y-axis in the bottom panel. Data are presented as mean± S.D. and significance is * P<0.05, **p<0.01, which was repeated for more than three times.


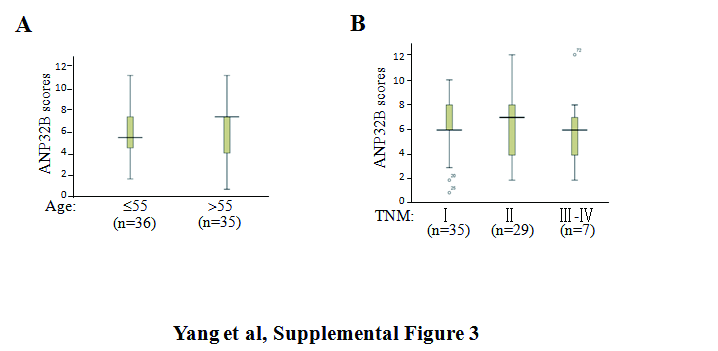


Figure S3, related to Figure 5

(A-B) Box plots of ANP32B expression in breast cancers patients with different age (A) and clinical stage (B). Data was analyzed by one-way ANOVA test.


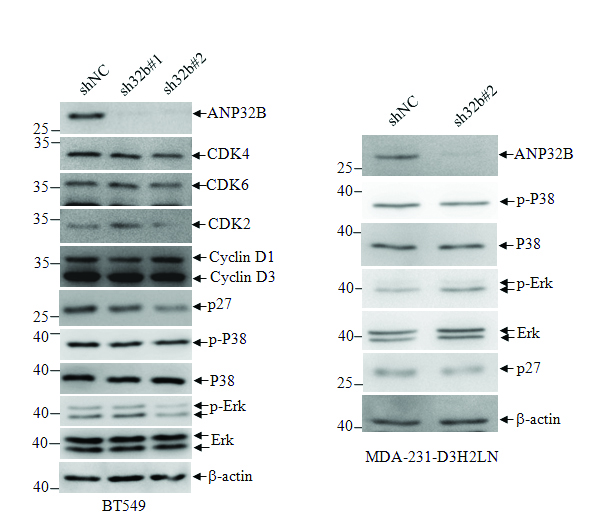


Figure S4, related to Figure 6

Western blots for the indicated protein in shNC and sh32b infected BT549 and MDA-231-D3H2LN cells.

**Supplemental Table S1, related to Figure 5.**

**Information of breast tumor and adjacent Samples**

| **No.** | **Tissues** | **Sex** | **Age** | **ANP32B scores** |
| --- | --- | --- | --- | --- |
| RRpBre0411A0019 | Tumor | F | 75 | 8.5 |
|  | Adjacent normal |  |  | 1.5 |
| CRpBre0412A0034 | Tumor | F | 50 | 10 |
|  | Adjacent normal |  |  | 6 |
| CRpBre0409A0013 | Tumor | F | 48 | 9 |
|  | Adjacent normal |  |  | 9 |
| RRpBre0411A0016 | Tumor | F | 52 | 10.5 |
|  | Adjacent normal |  |  | 3 |
| RRpBre0411A0017 | Tumor | F | 57 | 6 |
|  | Adjacent normal |  |  | 6 |
| CRpBre0409A0021 | Tumor | F | 53 | 10 |
|  | Adjacent normal |  |  | 6 |
| CRpBre0412A0022 | Tumor | F | 53 | 9 |
|  | Adjacent normal |  |  | 5 |
| CRpBre0412A0031 | Tumor | F | 42 | 9 |
|  | Adjacent normal |  |  | 6 |
| CRpBre0503A0035 | Tumor | F | 35 | 10 |
|  | Adjacent normal |  |  | 7.5 |
| RRpBre0504A0041 | Tumor | F | 52 | 10 |
|  | Adjacent normal |  |  | 3 |
| RRpBre0504A0043 | Tumor | F | 54 | 9.5 |
|  | Adjacent normal |  |  | 1.5 |
| RRpBre0504A0044 | Tumor | F | 48 | 8.5 |
|  | Adjacent normal |  |  | 5 |
| CRpBre0504A0045 | Tumor | F | 71 | 8.5 |
|  | Adjacent normal |  |  | 1.5 |
| CRpBre0504A0046 | Tumor | F | 48 | 4.5 |
|  | Adjacent normal |  |  | 3.5 |
| RRpBre0506A0048 | Tumor | F | 54 | 12 |
|  | Adjacent normal |  |  | 8.5 |
| RRpBre0506A0049 | Tumor | F | 52 | 8.5 |
|  | Adjacent normal |  |  | 2 |
| RRpBre0506A0050 | Tumor | F | 38 | 7 |
|  | Adjacent normal |  |  | 5 |
| CRpBre0506A0055 | Tumor | F | 45 | 4.5 |
|  | Adjacent normal |  |  | 3.5 |
| RRpBre0507A0065 | Tumor | F | 93 | 12 |
|  | Adjacent normal |  |  | 8.5 |
| RRpBre0507A0067 | Tumor | F | 47 | 8 |
|  | Adjacent normal |  |  | 2 |
| CRpBre0509A0068 | Tumor | F | 47 | 7.5 |
|  | Adjacent normal |  |  | 2 |
| RRpBre0511A0078 | Tumor | F | 80 | 6 |
|  | Adjacent normal |  |  | 2 |
| CRpBre0409A0011 | Tumor | F | 41 | 10 |
|  | Adjacent normal |  |  | 6.5 |
| RRpBre0609A0098 | Tumor | F | 40 | 9 |
|  | Adjacent normal |  |  | 7 |
| RRpBre0704A0120 | Tumor | F | 69 | 6 |
|  | Adjacent normal |  |  | 5.5 |
| RRpBre0704A0122 | Tumor | F | 80 | 8 |
|  | Adjacent normal |  |  | 4 |
| RRpBre0705A0128 | Tumor | F | 62 | 7.5 |
|  | Adjacent normal |  |  | 7.5 |
|  |  |  |  |  |
| RRpBre0709A0149 | Tumor | F | 44 | 9 |
|  | Adjacent normal |  |  | 6 |
| CRpBre0707A0161 | Tumor | F | 49 | 9 |
|  | Adjacent normal |  |  | 6.5 |
| CRpBre0711A0185 | Tumor | F | 61 | 6 |
|  | Adjacent normal |  |  | 6 |
| RRpBre0801A0190 | Tumor | F | 52 | 7 |
|  | Adjacent normal |  |  | 6 |
| RRpBre0802A0200 | Tumor | F | 60 | 10 |
|  | Adjacent normal |  |  | 4 |
| RRpBre0803A0205 | Tumor | F | 47 | 9 |
|  | Adjacent normal |  |  | 5 |
| CRpBre0801A0216 | Tumor | F | 53 | 8 |
|  | Adjacent normal |  |  | 6 |
| CRpBre0803A0217 | Tumor | F | 55 | 10.5 |
|  | Adjacent normal |  |  | 6 |
| RRpBre0806A0238 | Tumor | F | 58 | 9 |
|  | Adjacent normal |  |  | 3 |
| CRpBre0804A0241 | Tumor | F | 56 | 6.5 |
|  | Adjacent normal |  |  | 4 |
| RRpBre0808A0259 | Tumor | F | 57 | 8.5 |
|  | Adjacent normal |  |  | 4 |
| RRpBre0808A0264 | Tumor | F | 52 | 7.5 |
|  | Adjacent normal |  |  | 6 |
| RRpBre0809A0266 | Tumor | F | 54 | 9 |
|  | Adjacent normal |  |  | 6 |
| RRpBre0811A0283 | Tumor | F | 48 | 8.5 |
|  | Adjacent normal |  |  | 6 |
|  |  |  |  |  |
| RRpBre0812A0287 | Tumor | F | 46 | 10.5 |
|  | Adjacent normal |  |  | 7.5 |
| CRpBre0810A0298 | Tumor | F | 51 | 10 |
|  | Adjacent normal |  |  | 9 |
| CRpBre0811A0300 | Tumor | F | 35 | 7 |
|  | Adjacent normal |  |  | 5 |
| RRpBre0904A0339 | Tumor | F | 60 | 2.5 |
|  | Adjacent normal |  |  | 1.5 |
| RRpBre0803A0344 | Tumor | F | 63 | 10 |
|  | Adjacent normal |  |  | 7 |
| RRpBre0905A0377 | Tumor | F | 46 | 12 |
|  | Adjacent normal |  |  | 7.5 |
| RRpBre0906A0392 | Tumor | F | 54 | 8 |
|  | Adjacent normal |  |  | 5 |
| RRpBre0906A0395 | Tumor | F | 51 | 5 |
|  | Adjacent normal |  |  | 6 |
| RRpBre0907A0402 | Tumor | F | 46 | 12 |
|  | Adjacent normal |  |  | 11 |

**Supplemental Table S2, related to Figure 5.**

**Basic information of the patients with breast cancer.**

| **No.** | **Sex** | **Age** | **Histological grade** | **TNM stage** | **ANP32B scores** |
| --- | --- | --- | --- | --- | --- |
| 04-523 | F | 64 | 1 | Ⅲ | 4 |
| 05-250 | F | 82 | 1 | Ⅲ | 2 |
| 06-1748 | F | 44 | 1 | Ⅰ | 6 |
| 06-3183 | F | 50 | 1 | Ⅱ | 4 |
| 06-6022 | F | 78 | 1 | Ⅰ | 2 |
| 06-6839 | F | 73 | 1 | Ⅰ | 4 |
| 07-345 | F | 71 | 1 | Ⅰ | 1 |
| 08-5913 | F | 60 | 1 | Ⅰ | 8.5 |
| 08-658 | F | 52 | 1 | Ⅰ | 4 |
| 09-6487 | F | 72 | 1 | Ⅰ | 4 |
| 07-2345 | F | 69 | 1 | Ⅰ | 9 |
| 03-1023 | F | 43 | 2 | Ⅱ | 2 |
| 03-1985 | F | 48 | 2 | Ⅱ | 8 |
| 03-3561 | F | 51 | 2 | Ⅱ | 6 |
| 04-4342 | F | 49 | 2 | Ⅰ | 3 |
| 04-594 | F | 66 | 2 | Ⅱ | 8 |
| 04-6642 | F | 41 | 2 | Ⅰ | 8 |
| 04-92 | F | 41 | 2 | Ⅱ | 8 |
| 05-1250 | F | 43 | 2 | Ⅱ | 6 |
| 05-1538 | F | 44 | 2 | Ⅰ | 4 |
| 05-2448 | F | 70 | 2 | Ⅱ | 5 |
| 05-3981 | F | 77 | 2 | Ⅱ | 6 |
| 05-4157 | F | 48 | 2 | Ⅱ | 6 |
| 05-4211 | F | 48 | 2 | Ⅲ | 4 |
| 05-4370 | F | 53 | 2 | Ⅰ | 6 |
| 05-4843 | F | 46 | 2 | Ⅰ | 6 |
| 06-1505 | F | 77 | 2 | Ⅱ | 4 |
| 06-3356 | F | 58 | 2 | Ⅰ | 6 |
| 06-3669 | F | 73 | 2 | Ⅰ | 6 |
| 06-4147 | F | 65 | 2 | Ⅰ | 8 |
| 06-4189 | F | 52 | 2 | Ⅰ | 6 |
| 06-5425 | F | 53 | 2 | Ⅱ | 8 |
| 06-543 | F | 48 | 2 | Ⅰ | 6 |
| 06-5563 | F | 62 | 2 | Ⅰ | 8 |
| 06-6863 | F | 50 | 2 | Ⅰ | 6 |
| 06-788 | F | 47 | 2 | Ⅱ | 6 |
| 07-2663 | F | 58 | 2 | Ⅰ | 6 |
| 07-6273 | F | 60 | 2 | Ⅰ | 6 |
| 08-2388 | F | 70 | 2 | Ⅱ | 8 |
| 08-4657 | F | 57 | 2 | Ⅰ | 6 |
| 08-4765 | F | 44 | 2 | Ⅱ | 4 |
| 08-4917 | F | 37 | 2 | Ⅰ | 6 |
| 08-5237 | F | 56 | 2 | Ⅰ | 8 |
| 08-5516 | F | 56 | 2 | Ⅰ | 6 |
| 08-7235 | F | 76 | 2 | Ⅱ | 8 |
| 08-7414 | F | 52 | 2 | Ⅱ | 7 |
| 08-7758 | F | 78 | 2 | Ⅰ | 9 |
| 08-8068 | F | 51 | 2 | Ⅱ | 8 |
| 09-1047 | F | 55 | 2 | Ⅱ | 12 |
| 09-376 | F | 48 | 2 | Ⅱ | 3 |
| 09-4320 | F | 50 | 2 | Ⅰ | 8 |
| 03-128 | F | 46 | 3 | Ⅲ | 6 |
| 03-3283 | F | 48 | 3 | Ⅱ | 12 |
| 03-3822 | F | 55 | 3 | Ⅱ | 9 |
| 03-3823 | F | 51 | 3 | Ⅱ | 12 |
| 03-528 | F | 44 | 3 | Ⅳ | 6 |
| 05-3386 | F | 77 | 3 | Ⅱ | 4 |
| 05-4225 | F | 72 | 3 | Ⅰ | 8 |
| 05-5286 | F | 60 | 3 | Ⅱ | 8 |
| 05-6234 | F | 51 | 3 | Ⅰ | 4 |
| 06-1338 | F | 68 | 3 | Ⅱ | 4 |
| 06-283 | F | 47 | 3 | Ⅳ | 8 |
| 06-3123 | F | 66 | 3 | Ⅳ | 12 |
| 06-3641 | F | 44 | 3 | Ⅰ | 6 |
| 06-3884 | F | 53 | 3 | Ⅱ | 8 |
| 06-389 | F | 62 | 3 | Ⅱ | 8 |
| 06-3910 | F | 58 | 3 | Ⅰ | 10 |
| 07-2397 | F | 67 | 3 | Ⅱ | 12 |
| 09-1994 | F | 59 | 3 | Ⅰ | 8 |
| 09-3103 | F | 69 | 3 | Ⅰ | 8 |
| 09-3770 | F | 77 | 3 | Ⅰ | 10 |

**Supplemental Table S3, related to Figure 5.**

**Association between ANP32B and p-AKT expressions in breast cancer tissues.**

| **p-AKT**  **expression** | **Low ANP32B expression**  **n %** | | **High ANP32B expression** | |
| --- | --- | --- | --- | --- |
|  |  |  | **n** | **%** |
| **Low** | **85** | **87.6** | **11** | **27.5** |
| **High** | **12** | **12.4** | **29** | **72.5** |
| **Total** | **97** | **100** | **40** | **100** |

**Data were analyzed by Chi-square test, p<0.0001**
